# Recent advances in understanding liver fibrosis: bridging basic science and individualized treatment concepts

**DOI:** 10.12688/f1000research.14841.1

**Published:** 2018-06-27

**Authors:** Ralf Weiskirchen, Sabine Weiskirchen, Frank Tacke

**Affiliations:** 1 Institute of Molecular Pathobiochemistry, Experimental Gene Therapy and Clinical Chemistry (IFMPEGKC), RWTH University Hospital Aachen, Pauwelsstraße 30, Germany; 2 Department of Medicine III, RWTH University Hospital Aachen, D-52074 Aachen, Pauwelsstraße 30, Germany

**Keywords:** NASH, liver fibrosis, microbiota, therapy, genetic disorders, viral infection, hepatitis, steatosis, collagen.

## Abstract

Hepatic fibrosis is characterized by the formation and deposition of excess fibrous connective tissue, leading to progressive architectural tissue remodeling. Irrespective of the underlying noxious trigger, tissue damage induces an inflammatory response involving the local vascular system and the immune system and a systemic mobilization of endocrine and neurological mediators, ultimately leading to the activation of matrix-producing cell populations. Genetic disorders, chronic viral infection, alcohol abuse, autoimmune attacks, metabolic disorders, cholestasis, alterations in bile acid composition or concentration, venous obstruction, and parasite infections are well-established factors that predispose one to hepatic fibrosis. In addition, excess fat and other lipotoxic mediators provoking endoplasmic reticulum stress, alteration of mitochondrial function, oxidative stress, and modifications in the microbiota are associated with non-alcoholic fatty liver disease and, subsequently, the initiation and progression of hepatic fibrosis. Multidisciplinary panels of experts have developed practice guidelines, including recommendations of preferred therapeutic approaches to a specific cause of hepatic disease, stage of fibrosis, or occurring co-morbidities associated with ongoing loss of hepatic function. Here, we summarize the factors leading to liver fibrosis and the current concepts in anti-fibrotic therapies.

## Introduction

Hepatic fibrosis is a frequent and potentially life-threatening complication associated with most chronic liver diseases, thereby representing a high medical and economic burden. Although major advances in the molecular understanding of liver fibrosis were achieved during the last few decades in a wealth of experimental studies, the translation of this knowledge into clinical practice is still limited. On the one hand, this is somewhat surprising, since the molecular and cellular mechanisms contributing to the pathogenesis of fibrosis are well understood and evolutionarily conserved, independent of the disease-causing noxa. On the other hand, simple pharmacological intervention (fibroprevention, fibrostasis, and fibrolysis) with anti-fibrogenic compounds targeting the fibrogenic matrix is complicated and often effective only in experimental models. In addition, liver fibrosis is not a simple reaction but is the interplay of a multitude of different soluble mediators (cytokines and chemokines) and diverse liver-resident and infiltrating cellular subsets and is further modulated by the chemical and biological properties of the disease-causing agent. In the following, we will briefly summarize the changes induced by these factors and their contribution to the pathogenesis of hepatic fibrosis. In the second part of this synopsis, we will address current strategies in the clinical management of hepatic fibrosis.

## Pathogenesis of hepatic fibrosis

**Figure 1.**
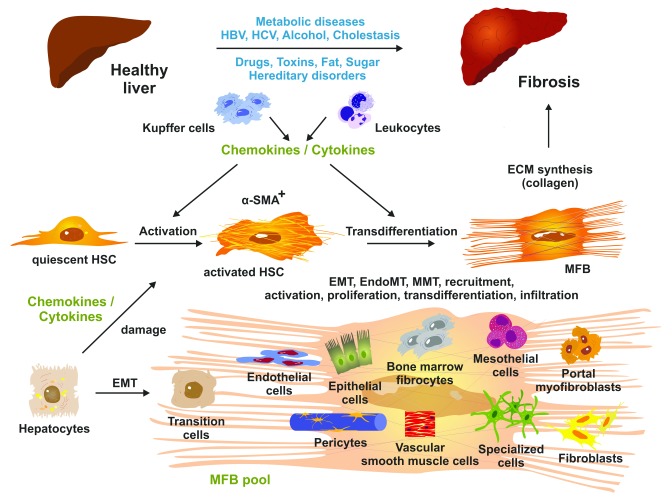
Pathogenesis of hepatic fibrosis. Prolonged liver injury results in changes in hepatic architecture and advanced fibrosis. At the cellular level, quiescent hepatic stellate cells (HSCs) become triggered by soluble mediators (chemokines and cytokines) released by liver-resident macrophages (Kupffer cells), infiltrating leukocytes, and other cell types including damaged hepatocytes. Both activated HSCs and transdifferentiated myofibroblasts (MFBs) are positive for α-smooth muscle actin (α-SMA). MFBs are the predominant source of collagen synthesis and deposition. The pool of extracellular matrix (ECM)-producing MFBs is further increased by different cell types such as resident fibroblasts, mesothelial cells, circulating (bone marrow) fibrocytes, epithelial cells, endothelial cells, pericytes, vascular smooth muscle cells, and other specialized cell types that acquire pro-fibrogenic activities and become capable of expressing ECM components. The relevant molecular and cellular mechanisms including epithelial-to-mesenchymal transition (EMT), endothelial-to-mesenchymal transition (EndoMT), mesothelial-to-mesenchymal transition (MMT), recruitment, activation, proliferation, transdifferentiation, and infiltration are being intensively studied presently. For more details, see 2, 15. HBV, hepatitis B virus; HCV, hepatitis C virus.

**Figure 2.**
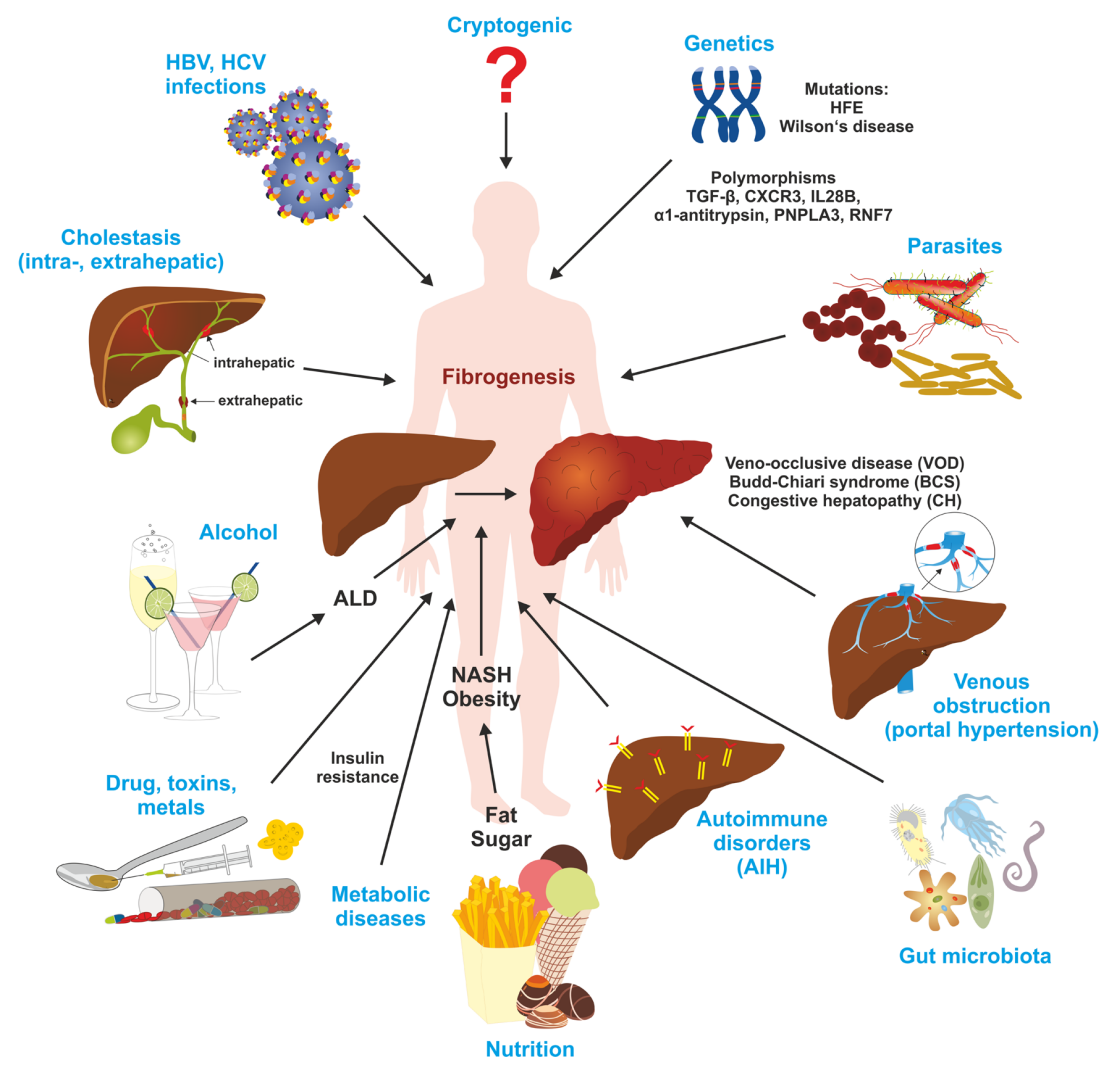
Major causes of hepatic fibrosis. In the liver, genetic alterations, metabolic disorders, cholestasis, viral infections, parasites, drugs, toxins, alcohol leading to alcoholic liver disease (ALD), and a wide variety of other noxious compounds and environmental factors can lead to the initiation and progression of fibrosis. AIH, autoimmune hepatitis; CXCR3, C-X-C motif chemokine receptor 3; HBV, hepatitis B virus; HCV, hepatitis C virus; IL28B, interleukin 28B; NASH, non-alcoholic steatohepatitis; PNPLA3, patatin-like phospholipase domain-containing protein 3; RNF7, ring finger protein 7; TGF-β, transforming growth factor-β.

The pathogenic sequence of fibrogenesis is initiated by parenchymal cell destruction resulting from a large variety of hepatotoxic and injurious agents and mechanisms. In most cases, the damage of tissue first induces an inflammatory response involving the local vascular system and the immune system and a systemic mobilization of endocrine and neurological mediators. In the mediation of this response, non-parenchymal cells (endothelium and stellate cells) and resident immune cells (macrophages, dendritic cells, and mast cells) possessing specialized surface receptors, which detect pathogen-associated molecular patterns (PAMPs) such as bacterial toxins and damage-associated molecular patterns (DAMPs), are triggered to release a large variety of different inflammatory and pro-fibrogenic mediators within the liver tissue. These lead to the activation of matrix-producing cell populations including hepatic stellate cells (HSCs) transiting to myofibroblasts (MFBs), portal MFBs, resident fibroblasts, and many other cell types contributing to the MFB pool ( Figure 1) ^1, 2^. Liver fibrosis can be a causative result of different underlying etiologies including genetic disorders, chronic viral infection, excessive alcohol consumption, autoimmune attacks, metabolic disorders, decreased bile flow, venous obstruction, and parasite infection ( Figure 2). Moreover, excessive lipids and other lipotoxic agents resulting in endoplasmic reticulum stress, alteration of mitochondrial function, oxidative stress in both parenchymal and non-parenchymal liver cells, and modifications in the microbiota composition of the gastrointestinal tract or its integrity are suggested to be associated with non-alcoholic fatty liver disease and hepatic fibrosis ^3^. The progression of hepatic disease is also triggered by alterations in bile acid composition. Bile acids are amphipathic molecules synthesized in the liver from cholesterol with manifold physiological functions. On the one hand, they facilitate the emulsification of dietary fats and assist the intestinal absorption of lipids and lipophilic vitamins ^4^. On the other hand, they act like hormones and are embedded in a complex network of signaling cascades. The most important targets of bile acids are the farnesoid X receptor (FXR) and the G-protein-coupled membrane receptor 5 (TGR5), which activate the expression of genes involved in the metabolism of bile acids, lipids, and carbohydrates ^5^. Importantly, bile acids also have antimicrobial activity that can damage bacterial cell membranes and thus inhibit bacterial overgrowth, protecting the liver and intestine against inflammation.

### Genetic disorders

Nowadays, a number of genetic diseases have been identified to directly or indirectly provoke hepatic fibrogenesis. The affected genes are involved in the control of metal homeostasis, bile acid synthesis or transport, carbohydrate metabolism, amino acid metabolism, the urea cycle, or the regulation of lipid metabolism ( Table 1). The prevalence, penetrance, age at onset, and disease outcome with regard to hepatic fibrosis can be significantly variable for the different triggers. Although metals play a host of pivotal roles in the liver by either being part of protein complexes or acting as essential cofactors and catalysts, elevated concentrations of metals result in intracellular reactive oxygen species (ROS) formation, induction of necrosis and apoptosis, and release or synthesis of pro-fibrogenic soluble mediators ^6^. Elevated iron concentrations are the characteristic result in hereditary hemochromatosis provoked by mutations within the *HFE* gene or other genes ( *SLC40A1*, *TFR2*, *HAMP*, and *HJV*) controlling iron homeostasis ^7^. Likewise, hepatic damage in Wilson's disease affecting the uptake and clearance of ingested copper is induced by mutations within the *ATP7B* gene ^8^. Genetic disorders associated with functional alterations of ATP-binding cassette (ABC) transporters utilizing the energy of ATP binding and hydrolysis in the participation of hepatobiliary transport of metabolites, phospholipids, and cholesterol and different bile acid translocation of various substrates across cellular membranes can provoke fibrosis when failing to protect the cells from endogenously produced toxic compounds and metabolites. These genetic disorders might provoke familial hypercholesterolemia ( *ABCG5* and *ABCG8*), progressive familial intrahepatic cholestasis type 3 ( *ABCB4*), cystic fibrosis liver disease ( *CFTR*), general hepatic manifestation, or injury of the hepatobiliary system ^9^. Moreover, mutations affecting genes involved in amino acid synthesis or catabolism including fumarylacetoacetate hydrolase 1 ( *FAH*), arginosuccinate lyase ( *ASL*), and solute carrier family 25 member 13 ( *SLC25A13)* are prone to induce hepatic fibrogenesis. Another group of genes associated with hepatic fibrogenesis contains metabolic genes affecting cholesterol metabolism ( *LIPA*) or fructose intolerance ( *ALDOB*), in which alterations can provoke liver fibrosis due to a massive accumulation of cholesteryl esters or sugars ^10– 12^. In α1-antitrypsin deficiency, point mutations are the cause of misfolded and polymerized protein accumulating in the endoplasmic reticulum of hepatocytes, causing liver disease and hepatic fibrosis in children and adults ^13, 14^.

**Table 1.** Selected inherited disorders associated with hepatic fibrosis.

Disease	Gene	OMIM/cytogenetic location	Prevalence *	Remarks	Reference
Wilson's disease	ATP7B	606882 / 13q14.3	1–9:100,000	ATPase, plasma membrane copper-transport protein	8
Hereditary hemochromatosis	HFE	613609 / 6p22.2	3–5:1,000	Membrane protein with similarity to major histocompatibility complex class I proteins; associates with transferrin receptor	7
Non-HFE hereditary hemochromatosis Ferroportin Transferrin receptor 2 Hepcidin Hemojuvelin	SLC40A1 TFR2 HAMP HJV	604653 / 2q32.2 604720 / 7q22.1 606464 / 19q13.12 608374 / 1q21.1	<1:1,000,000 <1:1,000,000 <1:1,000,000 <1:1,000,000	Transmembrane protein transporting cellular iron from the inside to the outside Involved in cellular uptake of transferrin-bound iron Key regulator of entry of iron into the circulation Co-receptor for bone morphogenetic proteins	7
Sitosterolemia / Hepatobiliary cholesterol transporter 5 and 8	ABCG5 ABCG8	605459 / 2p21 605460 / 2p21	1–9:1,000,000 1–9:1,000,000	ATP-binding cassette transporters encoding major susceptibility genes for gallstones	9
Progressive familial intrahepatic cholestasis type 3	ABCB4	171060 / 7q21.12	1–9:100,000	ATP-binding cassette transporters encoding a multidrug-resistance protein	28
Hereditary fructose intolerance	ALDOB	612724 / 9q31.1	1–9:100,000	Liver-type tetrameric aldolase involved in glycolysis and gluconeogenesis	12
Tyrosinemia type I	FAH	613871 / 15q25.1	1:100,000 (birth incidence)	Enzyme involved in catabolism of phenylalanine	10
Argininosuccinate lyase deficiency	ASL	608310 / 7q11.21	1–9:100,000	Enzyme catalyzing the production of arginine	11
Citrin deficiency	SLC25A13	603859 / 7q21.3	NN	Calcium-dependent mitochondrial solute transporter with a role in urea cycle function	29
Cholesteryl ester storage disease and Wolman disease	LIPA	613497 / 10q23.31	1–9:1,000,000	Enzyme involved in the preduodenal breakdown of ingested triglycerides	30
α-1 antitrypsin deficiency	SERPINA1	613497 / 10q23.31	1–5:10,000	Plasma serine protease inhibitor	14, 31
Cystic fibrosis	CFTR	602421 / 7q31.2	1–9:100,000	ATP-binding cassette transporter conducting chloride and thiocyanate ions across epithelial cell membranes	32
Alström syndrome	ALMS1	606844 / 2p13.1	1–9:1,000,000	Protein involved in ciliary function and structure maintenance	33
(Isolated) congenital hepatic fibrosis	NN	NN / NN	0.5–1:10,000	Developmental disorder of the portobiliary system characterized histologically by defective remodeling of the ductal plate, abnormal branching of the intrahepatic portal veins, and progressive fibrosis of the portal tracts; heredity: autosomal recessive, or X-linked, or autosomal dominant.	34

*Depicted frequencies were taken from Orphanet ( http://www.orpha.net/), GeneReviews ( https://www.ncbi.nlm.nih.gov/books/NBK1116/), or the National Organization of Rare Disorders ( https://rarediseases.org). NN, not known; OMIM, Online Mendelian Inheritance in Man.

### Cholestasis

Cholestatic liver disease and disruption of proper bile secretion leads to liver damage, inflammation, and fibrosis. Mechanistically, hepatic accumulation of bile compounds consisting of salts and strong detergents causes unspecific cellular damage and initiation of a cascade of inflammatory and fibrogenic events in the liver. Primary biliary cholangitis (PBC), primary sclerosing cholangitis (PSC), biliary atresia, or loss-of-function mutations such as those found in Alagille syndrome affecting the *JAG1* or *NOTCH2* genes are only some causes of impaired bile flow by the liver ^16^. Moreover, direct mechanical obstruction of the bile flow from the liver into the duodenum, termed extrahepatic cholestasis, caused by neoplastic invasion of the biliary tree (e.g. cancers of extrahepatic bile ducts, gallbladder, or ampulla of Vater), cysts, stones in the common bile duct, pancreatitis, or narrowing of the bile duct can provoke impairment of bile flow and induce hepatic fibrosis ^17^.

### Alcohol

An overwhelming number of patients with excessive alcohol consumption and alcoholic hepatitis present with marked fibrosis ^18^. In alcoholic liver disease, the fibrogenic response in the liver after the uptake of alcohol is driven by acetaldehyde. Acetaldehyde is the first metabolite during the detoxification of ethanol, which can upregulate the transcription of collagen I directly and indirectly by triggering the synthesis of transforming growth factor-β1 (TGF-β1) ^19^. Moreover, chronic intake of alcohol provokes ROS formation, increases the multiplication of intestinal bacteria, and changes the intestine’s permeability to macromolecules, thereby increasing gut-derived endotoxins in the portal circulation and activating Kupffer cells through the Toll-like receptor pathways ^20^.

### Hepatitis virus infection

Infections with hepatitis B or C viruses are a global health problem associated with significant mortality and account for more than 1.3 million deaths per year and highly variable regional incidence rates and gender susceptibility for resulting complications ^21– 23^. Both infections are characterized by persistent hepatic inflammation, representing an important driver in establishing fibrosis and cirrhosis. Earlier studies estimated that, globally, 57% of cirrhosis and 78% of hepatocellular carcinoma cases are attributable to chronic infection with either hepatitis B or hepatitis C ^24^. Antiviral treatment strategies and the general introduction of effective hepatitis B vaccination have reduced the burden of hepatitis B ^25^, while the introduction of direct antiviral agents against hepatitis C virus offers the option to cure the disease worldwide ^26^.

### Drugs

Drug-induced liver injury (DILI) is one of the leading causes of acute liver failure in the Western world, with paracetamol (acetaminophen [APAP]) being the commonest causative drug followed by antimicrobials ^27^. The pattern of DILI-related hepatotoxicity is classified as hepatocellular, cholestatic, or mixed type. DILI can, in rare cases, also result in fibrosis or other patterns of chronic injury such as nodular regenerative hyperplasia, vanishing bile duct syndrome, or even cirrhosis ^27^.

### Autoimmune disorders

Autoimmune diseases of the liver can affect either the liver parenchyma (autoimmune hepatitis [AIH]) or the bile ducts (PBC and PSC). The overwhelming immune reaction promotes progressive destruction of the parenchyma, further inflammation, loss of hepatic function, and fibrosis ^35^. Based on the type of autoantibodies generated, three types of autoimmune hepatitis are historically classified. Patients with type 1 AIH are characterized by the presence of anti-nuclear antibodies and/or antibodies directed against smooth muscle actin. Serum from type 2 AIH patients contains antibodies directed against microsomes of liver and kidney, and serum from type 3 AIH patients typically contains auto-antibodies directed against soluble liver antigen ^35^. In PBC, more than 60 auto-antibodies were identified from which the presence of anti-mitochondrial antibodies is pathognomonic, while anti-nuclear antibodies are found in approximately half of patients ^36^. Likewise, in PSC patients, a large variety of auto-antibodies were detected which react with epitopes present in the mitochondria or nuclei of biliary or colonic epithelial cells, neutrophil granules, or ubiquitously expressed compounds ^37^. However, the exact pathogenesis of PSC is largely obscure, and the disease is characterized by progressive fibrotic fibers around bile ducts, recurrent episodes of bacterial cholangitis, and a high risk for malignant transformation ^38^. Overlapping features between AIH and cholestatic disorders such as PBC, PSC, or indeterminate cholestasis, known as overlap syndromes, are not uncommon. The diagnosis of AIH and its overlap variants are based on an integrated analysis of symptoms, clinical findings, biochemical tests, serologic features, and liver histology ^39^.

### Nutrition and metabolic diseases

Over-nutrition, obesity, and its multiple metabolic sequelae, including type 2 diabetes mellitus, heart and blood vessel diseases, and fatty liver disease, are advancing worldwide, especially in the Western countries. Insulin resistance and hepatic fat deposition accompanied by steatosis, lipotoxicity, endoplasmic reticulum stress, parenchymal injury, and death are triggering hepatic inflammation, HSC activation, and progressive fibrogenesis ^40^. Recent evidence further suggests that diets enriched in sugar are also key in the pathogenesis of non-alcoholic fatty liver disease (NAFLD) and non-alcoholic steatohepatitis (NASH) by impacting gut microbiota and triggering hepatic fat accumulation due to the stimulation of lipogenesis and impairment of fat oxidation ^41, 42^. Contrarily, several bioactive food components such as caffeine, vitamins, curcumin, silymarin, resveratrol, quercetin, epigallocatechin-3-gallate, and many others are known to protect against hepatic fibrosis ^43, 44^. Recently, the gut microbiota has been considered as a key modulator of host metabolism. The gut microbiota not only facilitates the harvesting of nutrients and energy from ingested food but also is essential in the production of numerous metabolites necessary for proper host metabolism, including bile acids, which regulate diverse metabolic pathways in the host ^5^.

### Gut microbiota

The integrity of the intestinal barrier is highly crucial to the function of the gut–liver axis. A leaky gut and a pathological translocation of bacteria or bacterial components resulting from quantitative or qualitative changes in the gut microbiota (dysbiosis) lead to the activation of resident liver macrophages (i.e. Kupffer cells) releasing pro-inflammatory cytokines and stimulation of matrix synthesis by HSCs through Toll-like receptors ^45^. Changes in the gut microbiome detected in non-invasive, stool-based tests were recently shown to be clinically useful to diagnose metabolic diseases and advanced fibrosis in patients with NAFLD ^46^. In addition, there is a correlation among gut microbiota composition, alcohol, and ROS formation (see above).

### Venous obstruction

Obstruction of the portal venous flow can result from intrahepatic or extrahepatic portal vein thrombosis or portal cavernoma. General risk factors for portal vein thrombosis are reduced portal flow velocity, hypercoagulable tendency, vascular damage, and malignant vascular invasion ^47^. Hepatic venous outflow obstruction typically provokes sinusoidal congestion and parenchymal cell necrosis mostly in perivenular areas of hepatic acini, which may lead to the formation of bridging fibrosis between adjacent central veins ^48^. The disease is complex and the obstruction might occur in the small veins in the liver (veno-occlusive disease) or in the draining liver veins (Budd-Chiari syndrome) or is the result of increased pressure in the sublobular branches of hepatic veins due to chronic heart failure (congestive hepatopathy). When not properly treated, chronic obstruction leads to ischemic necrosis, subsequent collagen accumulation, and regional vein wall remodeling.

### Parasites

The most important parasitic diseases associated with liver fibrosis are schistosomiasis and echinococcosis. Hydatid cysts arising from *Echinococcus granulosus* or *Echinococcus multilocularis* and granulomas formed around trapped eggs during infection with *Schistosoma mansoni* and *Schistosoma japonicum* give rise to fibrosis around the trapped parasitic invader component resulting from host immune responses ^49^. Experimental studies have shown that hepatic hydatidosis due to *E. granulosus* infection results in increased TGF-β1 mRNA and protein expression in the middle and late stages of infection and stimulates the development of hepatic fibrosis ^50^. Similarly, a study analyzing 39 patients with cystic echinococcosis revealed that the TGF-β receptor II occupying a central role in the transduction of TGF-β1 signals was significantly upregulated in *E. granulosus*-infected areas when compared with the adjacent normal liver tissues ^51^.

### Cryptogenic and congenital liver disease

Typically, cryptogenic development of hepatic fibrosis and cirrhosis occurs in mid to late adulthood as a result of inherited disorders. In some cases, mutations in certain cytokeratin proteins (keratin 18 and keratin 8) are associated with the pathogenesis of cryptogenic and congenital liver disease. In this regard, conserved amino acids located at the junctures between individual structural motifs within keratins are most effective in producing hepatic fibrosis and cirrhosis by interfering with the normal reorganization of keratin filaments ^52^. Other reports suggested cryptogenic liver disease as a consequence of unidentified mutation within ABC transporters involved in limiting intestinal absorption and promoting biliary excretion of sterols, such as the *ABCG5* (Sterolin-1) and *ABCG8* (Sterolin-2) genes ^53^.

## Existing and emerging therapies for hepatic fibrosis

**Figure 3.**
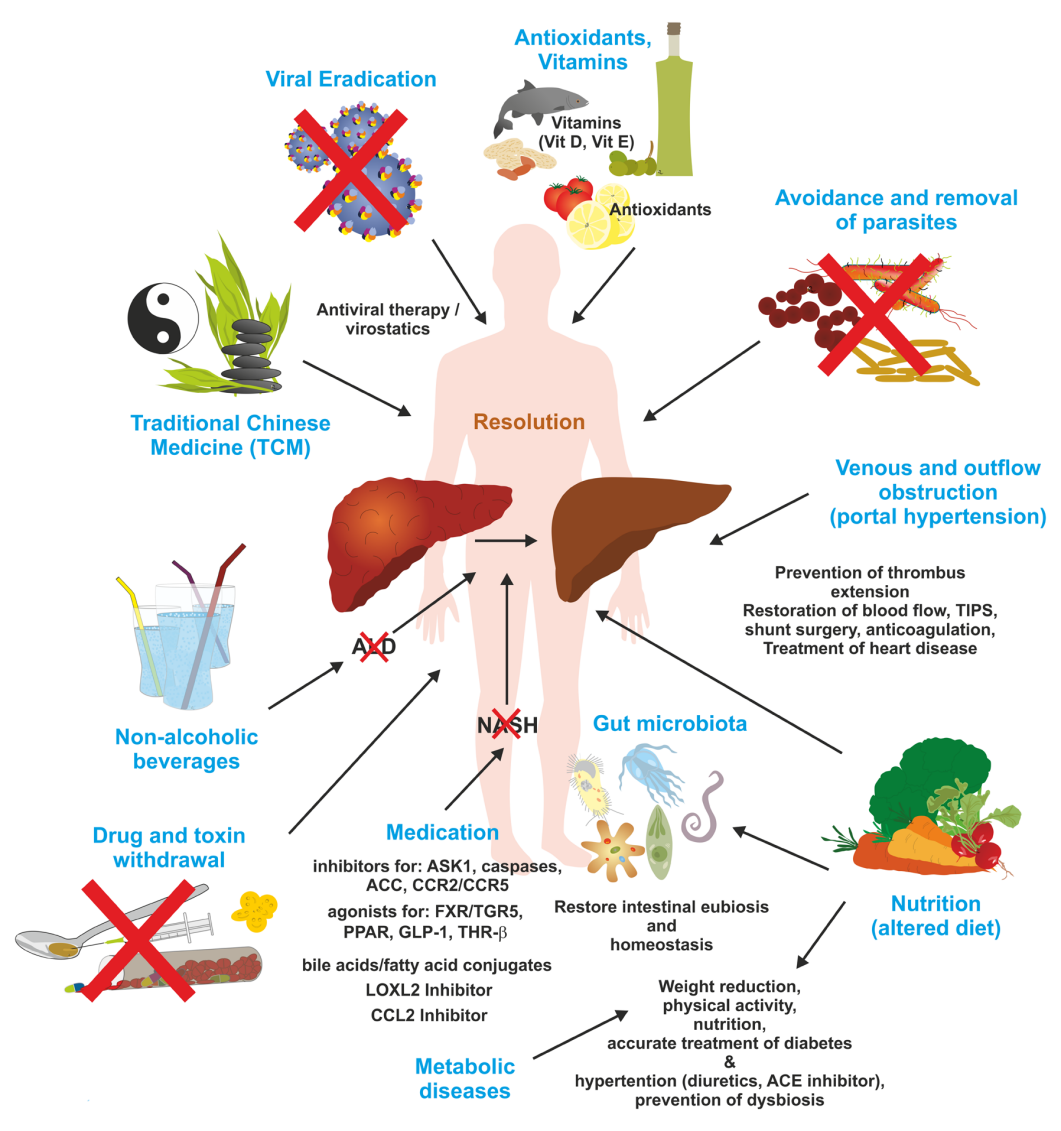
Strategies to induce resolution of hepatic fibrosis. Ongoing hepatic fibrosis can be haltered or resolved by specific therapies, eradication or dietary restriction of the pathogenic cause, or lifestyle and dietary interventions. In addition, several neglected features of lifestyle such as physical exercise, sun exposure, vitamin supplementation, and improved sleep duration and rhythm have been shown to be beneficial in the management of hepatic fibrosis. For more detailed explanations, see text. ACC, acetyl-CoA carboxylase; ACE, angiotensin-converting enzyme; ALD, alcoholic liver disease; ASK1, apoptosis signal-regulating kinase 1; CCL2, C-C motif chemokine ligand 2; CCR2/CCR5, C-C motif chemokine receptor 2/5; FXR, farnesoid X receptor; GLP-1, glucagon-like peptide-1; LOXL2, lysyl oxidase-like 2; NASH, non-alcoholic steatohepatitis; PPAR, peroxisome proliferator-activated receptor; TGR5, G-protein-coupled membrane receptor 5; THR-β, thyroid hormone receptor-β; TIPS, transjugular intrahepatic portosystemic shunt.

There are a large number of sound clinical practice guidelines and recommendations for the treatment of patients with hepatic fibrosis, according to the different types of liver diseases. Naturally, these guidelines focus on etiology-specific interventions or on the management of disease complications but do not recommend general “anti-fibrotic” therapies ( Table 2). The clinical treatment guidelines from professional associations such as the American Association for the Study of Liver Diseases (AASLD) or the European Association for the Study of the Liver (EASL) are important to improve the consistency and quality of care received by patients. In addition to these standardized guidelines, complementary and alternative therapies, herbal products, vitamins, or other dietary supplements are widely applied in patients suffering from chronic liver disease ( Figure 3). In the following, we will briefly sum up the current treatment regimens and the pathogenic basis of anti-fibrotic properties of specific treatments in hepatic fibrosis based on either experimental data or, in selected cases, clinical evidence.

**Table 2.** Selected strategies for treatment of hepatic fibrosis according to disease etiology.

Noxa	Treatment	Consequence of treatment	Reference
Hemochromatosis	Phlebotomy or chelating therapy	Reduction of iron content	54
Wilson's disease	Chelating therapy and zinc supplementation	Reduction of copper content and uptake	55
Sitosterolemia	Dietary restriction of cholesterol and plant sterols, sterol absorption inhibitors	Reduction of plasma plant sterols and cholesterol concentrations	9
Progressive familial intrahepatic cholestasis type 3	Nutritional support with calories, fat-soluble vitamins, and medium-chain triglycerides; medications to relieve pruritus; liver transplantation	Relief from pruritus, improvement of nutritional status, correction of vitamin deficiencies and treatment of complications (ascites and variceal bleeding) of advanced liver disease	28
Hereditary fructose intolerance	Dietary restriction of fructose, sucrose, and sorbitol¸ intravenous glucose (dextrose) administration, supportive treatment of hepatic insufficiency, and treatment of metabolic acidosis; supplementation with a "sugar-free" multivitamin	Reduction of toxic sugar effects, prevention of micronutrient deficiencies	56
Tyrosinemia type I	Dietary management with controlled intake of phenylalanine and tyrosine; medication with blockers of parahydroxyphenylpyruvic acid dioxygenase ( *p*-HPPD)	Prevention of the accumulation of fumarylacetoacetate and its conversion to succinylacetone	57
Argininosuccinate lyase deficiency	Control of hyperammonemia by discontinuing oral protein intake, supplementing oral intake with intravenous lipids and/or glucose, and use of intravenous arginine and nitrogen-scavenging therapy	Reduction and normalization of ammonia levels	58
Citrin deficiency	Supplement diet with fat-soluble vitamins and use of lactose-free and medium-chain triglyceride-enriched formula; administration of sodium pyruvate and arginine; liver transplantation	Prevention of hyperammonemic crises, correction of metabolic disturbances, and elimination of preferences for protein-rich foods	29
Cholesteryl ester storage disease and Wolman disease	Long-term enzyme replacement therapy with lysosomal acid lipase; hematopoietic stem cell transplantation	Correction of the metabolic defect	59
α-1 antitrypsin deficiency	Orthotopic liver transplantation; augmentation therapy (against pulmonary diseases); RNA interference-based therapeutics silencing the production of misfolded protein	Reduction of misfolded protein; increase of serum α-1 antitrypsin levels	14, 31
Cystic fibrosis	Optimization of nutritional state to avoid vitamin deficiency and malnutrition; medication with ursodeoxycholic acid	Stimulation of impaired biliary secretion and improvement of histological appearance	60, 61
Alström syndrome	Nicotinic acid derivatives for hyperlipidemia, lifestyle changes; individualized therapy	Lowering of lipids	62, 63
(Isolated) congenital hepatic fibrosis	No causative therapy available, symptomatic treatment	Lowering of persistent symptoms	34
Hepatitis C virus	Direct-acting antivirals	Sustained virologic responses (= cure)	64
Hepatitis B virus	Medication with nucleos(t)ide analogues	Viral suppression (possibly sustained hepatitis B surface antigen clearance or loss, representing “functional cure”)	65
Dysbiosis	Manipulation of the gut microbiota with diet, probiotics, or fecal microbiota transplantation	Growth promotion of "healthy" bacteria	66
Primary biliary cholangitis	Medication (ursodeoxycholic acid, obeticholic acid, budesonide, fibric acid derivatives)	Substitution of bile acids; cytoprotective effects in hepatocytes and cholangiocytes; regulation of genes involved in bile acid synthesis, secretion, transport, absorption, and detoxification; activation of peroxisome proliferator-activated receptors and downregulation of several pathways leading to bile acid synthesis	67
Primary sclerosing cholangitis	Medication (ursodeoxycholic acid); supportive treatment for symptoms; biliary dilatation (with or without stenting); liver transplantation	Lowered liver injury, relieving symptoms; reduction of portal hypertension	68
Alcohol	Abstinence/withdrawal, medical therapy of alcohol dependence; specific therapy of steatohepatitis; corticosteroids; liver transplantation	Restoration of liver architecture	69, 70
Drugs, toxins, and metals	Avoidance/abstinence/dose adjustment/withdrawal	Restoration of liver architecture	71
Autoimmune attack	Immunosuppressive treatment regimens (steroids, azathioprine, others); liver transplant	Lowering parenchymal destruction	72
Nutrition and metabolic diseases	Lifestyle modifications, mediation with insulin sensitizer agents, lipid- lowering drugs, antioxidants (vitamin E), statins	Lowering of fat uptake and cholesterol levels, normalization of blood glucose; lowering of oxidative stress	73
Venous obstruction	Anticoagulation (low-molecular-weight heparin), correction of risk factors, diuretics, prophylaxis for portal hypertension, angioplasty for short-length venous stenosis, transjugular intrahepatic portosystemic shunt, liver transplantation	Prevention of thrombosis and portal pressure	74
Parasites	Parasite-specific medication (e.g. praziquantel for all *Schistosoma* species)	Ensuring rapid and complete cure of infection and thus preventing the progression of hepatic disease	75
Cryptogenic and congenital liver disease	No specific therapy available, prevention of alcohol, obesity, diabetes mellitus, malnutrition, immunosuppressive agents, hepatotoxic medicines	Prevention of fibrosis progression	34

### Antioxidants

Oxidative stress and ROS are key drivers of hepatic inflammation and fibrosis. These are formed in the presence of elevated intracellular metal concentrations and also by the specific upregulation of different NADPH oxidases (NOXs) during liver fibrogenesis ^6, 43^. Therefore, there is great enthusiasm that antioxidants and radical scavengers targeting ROS are beneficial in the therapy of liver disease. Actually, some sulfur-containing and some non-sulfur-containing antioxidants have shown therapeutic effects in many experimental models of hepatic fibrosis ^43^. In addition, herbal ingredients (e.g. resveratrol, caffeine, and xanthohumol) and medicinal formulations and remedies originating from Traditional Chinese Medicine (TCM) such as *Sho-saiko-to* and *Salvia miltiorrhiza* have become the focus of global interest because these substances showed high anti-fibrotic capacity *in vitro* and in preclinical studies ^43^. Likewise, the herbal extract silymarin isolated from milk thistle containing the four main components silibinin, silidianin, silicristin, and isosilibinin has been suggested as an effective drug in experimental models that protects against major ROS and prevents mitochondrial failure and inflammation ^43^. In a randomized, placebo-controlled, multi-center prospective trial with 247 patients, high doses of the antioxidant vitamin E improved histological features of NASH over placebo but did not significantly reduce fibrosis ^76^. Thus, solid data on the clinical efficacy of antioxidants against fibrosis in humans are still limited.

### Inhibition of hepatic damage

In principle, anti-fibrotic drugs can mediate their effects on three different levels. A fibropreventive drug reduces liver cell damage by protecting hepatocytes from damage and enhancing the elimination of the noxious agent. Fibrostatic drugs suppress the production of new matrix by interfering with or blocking HSC activation or transdifferentiation. The last class consisting of fibrolytic drugs induces resolution of fibrosis by triggering necrosis and apoptosis of MFBs. Based on the complexity of hepatic fibrosis, the theoretical possibilities for therapeutic targeting are quite heterogeneous. Prevention of injury-mediated apoptosis/necrosis with the use of apoptosis blockers is an option addressing fibrosis initiation. Prototypically, the caspase inhibitor IDN-6556 (emricasan) was experimentally effective in protecting mice from cholestatic liver damage, inflammation, and hepatic fibrosis in mice ^77^. Likewise, the semi-synthetic bile acid obeticholic acid (OCA, INT-747) has anticholestatic and hepatoprotective properties, increases insulin sensitivity, modulates fat metabolism, and exerts anti-inflammatory and anti-fibrotic properties ^78^. Both agents are currently under clinical evaluation, and OCA has significantly reduced fibrosis in a clinical trial on patients with NASH ^79^. OCAs and similar drugs from this class target bile acid receptors like the FXR. Best known are endogenous (e.g. chenodeoxycholic acid, deoxycholic acid, cholic acid, and lithocholic acid) and (semi-)synthetic FXR agonists (OCA). In general, these components improve glucose metabolism, enhance insulin sensitivity, reduce hepatic lipogenesis, and increase β-oxidation ^4, 43^. Approved drugs that reduce intestinal cholesterol absorption, increase bile flow, change the hydrophobicity index of the bile acid pool, and provoke anti-inflammatory effects such as the steroid bile acid ursodeoxycholic acid (UDCA) or its side chain-shortened homologue norUDCA that has an increased cholehepatic shunting are also potential therapeutic alternatives ^80^.

### Targeting inflammation

Another therapeutic starting point is to target inflammatory mediators or to inhibit inflammatory monocyte infiltration. Neutralization of osteopontin becoming highly upregulated in fibrotic tissues, thereby increasing activity of TGF-β and chemoattraction of macrophages, was sufficient to abrogate fibrogenesis in mouse models of liver fibrosis by influencing liver progenitor cell function ^81^. Direct targeting of chemokines involved in the production of an inflammatory environment was also shown to arrest ongoing hepatic fibrogenesis ^82^. Prototypically, this was demonstrated by the application of an RNA-L-aptamer-based inhibitor (NOX-E36) targeting the CCL2/CCR2 axis. NOX-E36 is a synthetic 40mer mirror-image RNA L-aptamer (Spiegelmer), which potently binds to its pharmacologically relevant target molecule (i.e. CCL2) in a manner conceptually similar to an antibody and inhibits its biological activity. This drug prevented the attraction of inflammatory monocytes into the injured liver and ameliorated hepatic steatosis in murine models of acute and chronic hepatic injury ^83^. Likewise, transgenic overexpression or application of IL-22 induced HSC senescence, thereby reducing liver fibrosis, most likely by pathways involving STAT3, p53, and p21 ^84^. Encouraging pilot studies have shown that the serum amyloid P component (SAP) and other classical plasma pentraxin proteins bind directly to monocytes, neutrophils, and macrophages, thereby modifying their activation, spreading, and polarization and inhibiting their differentiation into fibrocytes ^85, 86^. It is obvious that these findings will trigger future studies aiming to investigate the beneficial effects of pentraxins in the attenuation of hepatic inflammation and fibrosis.

### Deactivation and elimination of extracellular matrix-producing cells

A main challenge in the attenuation of hepatic fibrosis is the lowering of cellular amount and activity of matrix-producing cells in the injured tissue. Targeted induction of apoptosis and senescence or the deactivation of the respective cell pool is experimentally achieved in many ways. Expression of proteins acting as a trigger of cellular senescence was capable of reducing the production of collagen type I at both mRNA and protein levels in HSCs and portal MFBs through the induction of ROS and attenuated TGF-β signaling ^87, 88^. Also, modulating the inflammatory environment can induce deactivation of matrix-producing MFBs ^89^.

### Inhibitors of cytokine signaling

There are numerous approaches demonstrating the efficacy of biological sequestering of pro-fibrogenic cytokines acting in an autocrine or paracrine manner. In particular, prominent anti-fibrotic effects were reported when targeting the TGF-β axis with its receptors and intracellular signaling mediators, the Smad proteins. Based on the complexity of TGF-β and its different signaling branches, the therapeutic targets are manifold. In experimental studies, soluble receptors biologically sequestering active TGF-β, dominant-negative TGF-β receptors, antisense strategies interfering with the synthesis of TGF-β, the expression of inhibitory Smad proteins, and the application of novel small inhibitors blocking the activity of individual receptors were successfully tested in preclinical models ^43^. Correspondingly, numerous investigations have demonstrated the applicability of anti-fibrotic strategies when targeting the platelet-derived growth factor (PDGF) family and its signaling compounds ^90^. This cytokine family has important roles in HSC and portal MFB proliferation, chemotaxis, migration, and cell survival. Respective therapeutic drugs (e.g. multikinase inhibitors and aptamers) targeting PDGF signaling are explored in advanced preclinical studies. Also, some cytokines developing inflammatory activity (TNF-α and interleukins) and apoptotic features (NGF), increasing portal pressure (VEGF), promoting fibrosis (FGF), or having immunomodulatory activity (IFN) or other pleiotropic activities influencing hepatic fibrogenesis (HGF and IGF) are under thorough investigation. However, although the list of potential anti-fibrotic drugs is rapidly increasing, most of these drugs have so far been tested only in experimental models of hepatic fibrosis and wait to be translated into clinical trials ^43^.

### Production and degradation of extracellular matrix

Fundamental work has shown that a balance between matrix metalloproteinases (MMPs) and their tissue inhibitors of metalloproteinases (TIMPs) is of fundamental importance in the homeostasis of extracellular matrix content within the liver ^91^. Moreover, the distinct expression and activity of individual MMPs and TIMPs are necessary to guarantee fibrolysis during the resolution of liver fibrosis ^92^. This knowledge will hopefully lead to the identification of novel therapeutic drug targets. Similarly, the lysyl oxidase-like 2 (LOXL2) belonging to a family of five proteins involved in collagen crosslinking and stabilization is key in promoting liver fibrosis and limiting its resolution. In line with this functionality, it was demonstrated that early treatment with the inhibitory monoclonal LOXL2 antibody AB0023 was effective in a mouse model of mild liver fibrosis by reducing the number of activated fibroblasts and decreasing the production of growth factors and cytokines ^93^. In a more recent study, this antibody was also shown to be effective in advanced, pre-established biliary and non-biliary fibrosis and in promoting the reversal of advanced parenchymal liver fibrosis in mice ^94^. Although these encouraging studies suggest that targeted LOXL2 inhibition is an attractive therapeutic option in fibrosis prevention or regression, clinical trials with the anti-LOXL2 antibody simtuzumab showed no anti-fibrotic activity in patients with hepatic fibrosis ^46^.

### Viral eradication, removal of parasites, and drug/toxin withdrawal

Numerous studies have confirmed that the simple withdrawal or suppression of the injurious stimulus can lead to the spontaneous resolution of fibrosis with a restoration to normal liver architecture ^95^. All these studies demonstrate that liver fibrosis is a dynamic bidirectional process, allowing regression and recovery even when fibrosis is advanced ^96^. Therefore, it is evident that the elimination or reduction of viral load in chronic viral hepatitis by anti-viral drugs (or combinations), the removal of hepatic parasites, or the withdrawal of any drug or toxin leading to parenchymal damage is still the first option in treating all fibrotic lesions of the liver. In most cases of parasitic infections, adequate medication (e.g. praziquantel for all *Schistosoma* species) is available.

### Restoration of intestinal eubiosis and homeostasis

The microbial ecosystem of the gastrointestinal tract is composed of a great number of highly varied microbial species. The sum and composition of these microorganisms, i.e. the microbiota, are critically influenced by the host’s genotype and environmental factors such as age, diet, drug use, alcohol intake, stress, and others. Intestinal eubiosis is important to guarantee proper intestinal functions including motility, energy harvest, mucosal immunity, and proper digestion. Modulation of gut microbiota by fecal transplant, also known as stool transplant, from a healthy individual into a recipient suffering from a disease presumed to be caused by alterations or disruption of the microbiota has become increasingly popular. Experimental work has demonstrated that the restoration of a healthy intestinal microbiota is sufficient to normalize portal hypertension in a rat model of NASH ^97^. Likewise, the manipulation of the intestinal microbiota by fecal microbiota transplantation with fresh feces from alcohol-resistant donor mice to alcohol-sensitive receiver mice prevented alcohol-induced liver injury, steatosis, and liver inflammation and restored gut homeostasis in mice ^98^. It is therefore conceivable that microbiota manipulation with anti-fibrotic microbes may allow the establishment of novel treatment possibilities in human liver disease.

### Restoration of blood flow

Portal vein thrombosis and venous obstruction are common complications in advanced liver fibrosis and cirrhosis. In patients with a persistent documented prothrombotic state, anticoagulation (e.g. with low-molecular-weight heparin) has been shown to prevent the progression of portal vein obstruction occurring because of thrombosis ^47^. Experimental models yielded divergent results on whether therapeutic anticoagulation (e.g. enoxaparin) reduces fibrosis and vascular resistance ^99, 100^. In a prospective randomized trial, enoxaparin prevented portal vein thrombosis, hepatic decompensation, and mortality in patients with advanced liver cirrhosis ^101^.

### Treatment of non-alcoholic steatohepatitis and non-alcoholic fatty liver disease

In the general population, NAFLD is estimated to afflict up to 30% of the general population in the Western countries and is in most cases provoked by physical inactivity and excessive calorie intake ^102^. In particular, NAFLD occurs in middle-aged subjects in association with obesity, hyperglycemia, dyslipidemia, and type 2 diabetes. The abundant fat tissue in visceral obesity is linked to enhanced secretion of pro-inflammatory adipokines including TNF, leptin, IL-6 and IL-18, RBP-4, CCL2, CXCL5, lipocalin 2 (LCN2), and ANGPTL2 ^103^. Therefore, the risk of NAFLD, NASH, diabetes, and associated metabolic syndrome could be principally prevented by lifestyle changes (healthy diet, weight loss, and regular physical exercise) that have direct consequences on inflammatory signaling pathways triggered by excess free fatty acids in obesity ^104^. However, the great wealth of tempting high-fat and high-sugar food propagated by industry and the hectic and stressful nature of today's Western world often hamper these adjustments. Therefore, several supportive pharmaceuticals are under close investigation. A recent meta-analysis of eight randomized clinical trials enrolling 516 patients with biopsy-proven NASH revealed that thiazolidinedione therapy (rosiglitazone or pioglitazone) was associated with improved fibrosis and NASH resolution, even in patients without diabetes ^105^. Other clinical candidate drugs for NASH are agonists for the FXR such as OCA (INT-747) or for the glucagon-like peptide-1 receptor (GLP-1R). Based on the beneficial effect of OCA, this compound is being tested presently (in phase 2 and phase 3 clinical studies) as a possible treatment for NASH, NAFLD, obesity, alcoholic hepatitis, and other liver and gastrointestinal conditions. The stimulation of FXR involved in the regulation of genes associated with bile acid synthesis and lipoprotein metabolism with the agonist WAY-362450 (turofexorate isopropyl, XL335, FXR-450) protected against hepatic inflammation and fibrosis in a murine model of NASH ^106^. In similar experimental studies in mice, GLP-1R agonists reduced body weight, liver mass, liver lipid, plasma alanine aminotransferase, and triglycerides ^107^. More recent findings suggested FXR and GLP-1 co-activation to be more therapeutically beneficial ^108^. Another NAFLD/NASH treatment option is the supplementation with vitamin E, which has shown beneficial effects on serum liver enzymes, steatosis, inflammation, hepatocellular ballooning, and hepatic fibrosis ^109^. In addition to these drugs, several anti-inflammatory drugs are under consideration. The apoptosis signal-regulating kinase 1 (ASK1) inhibitor selonsertib (GS-4997) has demonstrated reduced liver fibrosis, liver stiffness, and lobular inflammation in NASH patients and subjects with stage 2–3 fibrosis ^110^. Moreover, the PPARα/d agonist elafibranor improved inflammation and fibrosis in mouse models of NAFLD and has now entered phase 3 clinical trials ^111^. Similar to its therapeutic effects in experimental studies ^112, 113^, the C-C motif chemokine receptor 2/5 (CCR2/5) antagonist cenicriviroc (CVC) improved hepatic fibrosis and systemic inflammation in NASH patients ^114^. To sum up, there is a large list of compounds currently being evaluated in advanced clinical trials for the treatment of NASH and NAFLD. It is suggested that the availability of therapeutic options for NASH will curb the rising trend of respective diseases ^111^. Currently, other pharmacotherapeutics for NAFLD and NASH such as GR-MD-02, which binds to the carbohydrate-binding domain of galectin-3, which is upregulated in cases of liver fibrosis and has an essential function in cell–cell interactions, angiogenesis, apoptosis, and macrophage activation, are also under investigation ^115^. Beside these pharmaceutical treatment options, weight loss induced by lifestyle changes and controlled exercise have outstanding value in the treatment of fatty liver disease ^102^. This was impressively demonstrated in a prospective study of 293 patients with histologically proven NASH, in which effective lifestyle changes to reduce weight over 52 weeks improved histologic features associated with NASH ^116^.

## Conclusions

There is a large variety of factors, including genetic disorders, chronic viral infection, alcohol abuse, autoimmune attacks, metabolic disorder, cholestasis, venous obstruction, and parasite infection, that cause hepatic fibrosis. Although the molecular and cellular processes leading to fibrosis are more or less the same, the different etiologies require different treatments. Sound clinical practice guidelines and recommendations were established for almost any disease constellation. Beside standardized medication and individualized interventional strategies, many experimental studies are currently being conducted to test drugs that inhibit liver damage, target inflammation, reduce the number or activation state of matrix-producing cells, or inhibit the biological activity of pro-fibrotic soluble mediators (cytokines and chemokines). In particular, novel promising NASH and NAFLD drugs (GLP-1R antagonists, pioglitazone, vitamin E, elafibranor, OCA, selonsertib, simtuzumab, GR-MD-02, and CVC) from the pipeline of the pharmaceutical industry are being tested in advanced clinical trials and possibly will be approved as medications in the near future. However, in the case of NAFLD and NASH, lifestyle modification including specialized diets and physical activity leading to weight reduction, reduced liver fat, and improved glucose balance is still an effective option to improve liver overall health.

## Abbreviations

ABC, ATP-binding cassette; AIH, autoimmune hepatitis; CVC, cenicriviroc; DILI, drug-induced liver injury; GLP-1, glucagon-like peptide-1; GLP-1R, glucagon-like peptide-1 receptor; HCC, hepatocellular carcinoma; HSC, hepatic stellate cell; LOXL2, lysyl oxidase-like 2; MFB, myofibroblast; MMP, matrix metalloproteinase; NAFLD, non-alcoholic fatty liver disease; NASH, non-alcoholic steatohepatitis; PBC, primary biliary cholangitis; PDGF, platelet-derived growth factor; PSC, primary sclerosing cholangitis; ROS, reactive oxygen species; TGF-β, transforming growth factor-β; TIMP, tissue inhibitors of metalloproteinase.
